# Is ‘viability’ viable? Abortion, conceptual confusion and the law in England and Wales and the United States

**DOI:** 10.1093/jlb/lsaa059

**Published:** 2020-10-09

**Authors:** Elizabeth Chloe Romanis

**Affiliations:** Department of Law, Centre for Social Ethics and Policy, University of Manchester, Manchester M13 9PL

**Keywords:** abortion, artificial wombs, comparative law, criminal law, reproductive health, viability

## Abstract

In this paper, I explore how viability, meaning the ability of the fetus to survive post-delivery, features in the law regulating abortion provision in England and Wales and the USA. I demonstrate that viability is formalized differently in the criminal law in England and Wales and the USA, such that it is quantified and defined differently. I consider how the law might be applied to the examples of artificial womb technology and anencephalic fetuses. I conclude that there is incoherence in the meaning of viability and argue that it is thus a conceptually illegitimate basis on which to ground abortion regulation. This is both because of the fluidity of the concept and because how it has been thus far understood in the law is unsupported by medical realities. Furthermore, it has the effect of heavily diluting pregnant people’s rights with overly moralistic limitations on access to healthcare.

## I. INTRODUCTION

The status of human entities before birth has been a subject of debate for centuries and remains highly contested. In need of a pragmatic answer, the law in many jurisdictions has adopted a gradualist approach to abortion regulation and provision. Viability has, thus, become an important feature of the law. Viability is ‘the ability [of a developing fetus] to survive independent of a pregnant woman’s womb’.^1^ In jurisdictions adopting this approach, abortion is lawful or accessed with less difficulty, before the point that a fetus is deemed viable. After viability, abortion is lawful in a narrower set of circumstances and/or harder to access. Gradualism as grounds for determining the moral permissibility of abortion has been subject to heavy challenge in the philosophical literature. Viability remains, however, enshrined as a legal concept in multiple jurisdictions, perhaps exemplifying the gap between moral reasoning and the law (which is inevitably more arbitrary). Lawmakers often paint viability as a workable position somewhere between the demands of the anti-choice and the pro-choice campaigns found in the political landscape of England and Wales and the USA. The politicized labeling of viability as a compromise is misplaced, however, because viability limits access to healthcare. The meaning afforded to the concept of viability in the law is therefore hugely impactful.

In this paper, I consider how viability features, is defined, and is utilized in the law in England and Wales and the USA and whether these conceptions of viability are legally coherent. The impact of viability as a tool in legal regulation that limits access has been well explored. It is often argued that viability should not be utilized as a legal metric to ground abortion rights.^2^ In this paper, I consider the parameters of viability as a legal concept, specifically how it has been both defined and quantified and its conceptual grounding. Most literature that criticizes the concept has not considered the mechanics of the concept in terms of how it is defined and instrumentalized. Since viability is so entrenched in the law, we should have a clear understanding of its meaning that can be consistently articulated and be able to justify its inclusion as a legal standard. I demonstrate that while viability is often described in a simple account of ‘the ability of a fetus to survive after pregnancy’, the concept has been deployed, understood, and defined in vastly different ways in the USA and England and Wales. I argue that for viability to be considered a coherent concept, it must have a rational and cogent basis that is consistent about what kind of life *ex utero* matters legally and can be consistently applied in different circumstances. I echo calls made by other scholars that it is inappropriate to ground regulation by reference to viability; however, I do so by considering by what logics the particular legal regimes identified recognize viability as a justifiable concept and standard of compromise in abortion law.

I compare the law in these two jurisdictions because, despite some economic and cultural similarities between them, they have different modern histories of abortion law, abortion politics, and systems of provision.^3^ In the USA, abortion provision is in the hands of State legislatures that are subject to some restrictions by the Supreme Court, which has interpreted the Constitution, most notably in *Roe v. Wade*^4^ and *Planned Parenthood v. Casey*,^5^ to recognize that abortion is encompassed in the right to privacy. In England and Wales, no right to abortion is recognized, but Parliament has legislated to ensure medicalized access under certain circumstances.^6^ Viability features in both these frameworks to differing degrees. The US Supreme Court has been explicit that the right to abortion is limited by foetal viability. In England and Wales, the statutory framework has, both directly and indirectly, the effect of ensuring access becomes more limited after foetal viability. These jurisdictions are therefore useful points of comparison for examining the coherence of viability because it is featured, defined, and quantified differently in both.^7^

First, I demonstrate the extent to which viability determines the legality, or provision, of abortion in both jurisdictions. Second, I test the concept of viability that is contained in the law of each jurisdiction by considering its application to two specific cases: the development of artificial womb technology and anencephaly. Artificial womb technology is of interest because such a development might challenge the viability timeline by enabling the support of fetuses *ex utero* much earlier in gestation.^8^ Fetuses with conditions incompatible with life such as anencephaly are of interest because they allow investigation of the possibility that viability is a rebuttable presumption later in gestation. I demonstrate that there are internal inconsistencies in the application of the law in these jurisdictions in how the law is applied to these two case studies. There are, therefore, problems in how viability has been legally conceptualized and in implementing viability as a feature of the law, resulting in inconsistency in the legal recognition afforded to fetuses and in determining what kind of life matters *ex utero*. Finally, I argue that viability is not a conceptually legitimate basis for abortion regulation.

## II. THE ORIGINS OF VIABILITY

Before delving into the particularities of how viability is both featured and articulated in the law in the USA and England and Wales, it is necessary to first explore the conceptual foundations of viability as a concept.

The central role of gradualism in theorizing about the fetus has long been evident. Based on the work of physicians such as Hippocrates, who advanced a gradualist account of fetal formation and development,^9^ Aristotle considered the moral significance of a fetus as increasing with its development. His theory, centered on ‘ensoulment’, suggested that the embryo is a ‘vegetative soul’ potentially equivalent to that of plant life, but it attains a more sentient existence and soul as it attains a human shape—when the fetal body is formed and movement can be perceived.^10^ The theory that ‘quickening,’ the moment that fetal movement is first perceived, marked a morally significant moment in fetal development persisted throughout the centuries featuring in the work of Thomas Aquinas^11^ among others. These accounts focus, much like the modern conception of viability, on identifying a particular feature of the fetus’s capacities in order to isolate the point at which it can be thought of as a ‘living being’. The focus began shifting away from quickening to viability in the late 19th century with advancing medical technology and increasing obstetrical knowledge. Viability as a concept has its origins in the emergence of contemporary prenatal care. With the revelation of a design of a neonatal incubator at the World Exposition in 1896,^12^ viability became an increasingly important indicator as a matter of medical ‘pragmatic concern’^13^ in the context of prenatal care. Viability is, in a medical sense, the point at which there is a reasonable probability that a fetus could survive *ex utero* when provided with care. Primarily, the concept is utilized by clinicians in the context of spontaneous preterm delivery to determine whether the provision of intensive care is appropriate.^14^

There is some extent to which the theory that viability as the point at which the fetus becomes deserving of some protection as a ‘living being’, and thus is a relevant consideration in the regulation of abortion, came to fruition as a form of political compromise. Lavi explains that this is because the concept is often presented as a ‘plain reality and a non-controversial development stage that any reasonable person would acknowledge’.^15^ The logic being that ‘once the fetus is viable, its status as a full-human being seems to be a self-evident truth. The viable fetus no longer needs to become a full-human being; it is ready to begin to live an independent life’.^16^ It therefore, seemingly, presents a position based on an exercise of logic, but that also allows pregnant people the chance to access abortion care up to a certain point, after which the fetus is ‘entitled’ to certain protections. Later in this paper, I will review the significant criticism that is deployed against viability as a morally significant moment in fetal development and criticize the extent to which viability can be understood as a compromise in abortion regulation.

## III. VIABILITY IN THE LAW

This section outlines how abortion provision is dependent upon determinations about viability in England and Wales and the USA and makes some preliminary comparative observations.

### A. England and Wales

Under S.58 and 59 of the Offences against the Persons Act 1861, it is a crime for any person (including the pregnant person) to unlawfully procure a miscarriage. This crime can be committed at any point in gestation, irrespective of any perceived (in)capacities of the fetus to survive *ex utero*.^17^ The Infant Life (Preservation) Act 1929 created the offence of child destruction, committed when a person wilfully acts to intentionally destroy a fetus capable of being born alive.^18^ The Abortion Act 1967 (AA 1967) provides medical practitioners with a defence to these criminal offences in a proscriptive list of circumstances, the first of which is termed the ‘social ground for abortion’ and stipulates that termination is lawful if two doctors agree that ‘the pregnancy has not exceeded its 24 weeks and continuing the pregnancy would involve risk (greater than if the pregnancy were terminated) of injury to the physical or mental health of the pregnant woman or a existing children of her family’.^19^ This does not provide pregnant persons with a right to pregnancy termination, because the AA 1967 is still framed in medical terms; termination can be provided before 24 weeks provided that the risk of continuing the pregnancy is greater than the risks of termination. However, this provision is so broad it renders ‘every pregnancy legally terminable within the first 24 weeks’.^20^ This defence in the AA 1967 has installed an implicit viability threshold. There are defences available for doctors performing abortion after 24 weeks; however, they are harder to establish and require ‘clear proof of the more serious danger specified’.^21^ These grounds consist only of where the termination is necessary to prevent ‘grave permanent injury’ to the physical or mental health of the pregnant woman, where pregnancy is a greater risk to the woman’s life than termination, or where there is a substantial risk that the fetus has serious abnormalities.^22^ Because the defences available to a doctor to avoid criminal sanction after 24 weeks are narrower and framed in terms of greater severity, the AA 1967 appears to grant a limited ‘right to be gestated’ to fetuses from 24 weeks. This is, of course, subordinate to pregnant people’s life and health, but notably the law distinguishes between the justifications for abortion before and after this point.

There is a more explicit reference to viability in the offence of child destruction criminalizing the abortion of fetuses capable of being born alive*.* The Infant Life (Preservation) Act 1929 (ILPA 1929) provides limited guidance on the meaning of capable of being born alive, other than to stipulate that reaching 28 weeks’ gestation was prima facie proof. This minimum threshold was subsequently lowered to 24 weeks.^23^ Section 5 of the AA 1967 was amended in 1990^24^ to specify that this offence is not committed when a medical practitioner conducts abortion in the circumstances outlined by the Abortion Act. It remains unclear whether the Abortion Act offers the more stringent protection to fetuses only at 24 weeks and beyond, or whether fetuses before 24 weeks are also encompassed if proof can be if they are capable of being born alive*.* It is possible to view the 24-week threshold as either fixing legal viability to this point (regardless of whether this matches medical reality/opinion) or as a guideline marking the point from which viability is legally assumed, but not necessarily precluding the recognition of younger fetuses as viable.

The language used in the AA 1967 and ILPA 1929 is significant. There were ample opportunities for Parliament when drafting the AA 1967, or amending it in 1990, to specify that the AA 1967 24-week threshold was superseding a ‘capable of being born alive’ standard. It plausible, therefore, that capable of being born alive was intended to afford protection to some fetuses before 24 weeks; preventing termination unless the grounds under the AA 1967 were met. Judges have interpreted ‘capable of being born alive’ in this way. In *C v. S*,^25^ the Court of Appeal judgment afforded considerable time to considering medical evidence to ascertain whether an 18-week fetus was capable of being born alive under the ILPA 1929. The legislative threshold is thus ‘only a presumption which relieves the prosecution of the burden of proving viability’ after that point and ‘does not prevent proof that a particular fetus is viable at an earlier stage in development’.^26^  *C v. S* was decided before the 1990 amendments to the AA 1967 but demonstrates that there is potential for the viability timeline to be shifted, increasing the scope for liability if there is not strict adherence to the defences in the AA 1967.

What is encompassed in the capacity to be born alive? In *C v. S*, Donaldson MR stipulated that a fetus was capable of being born alive only if it could breathe after birth, with or without a ventilator.^27^ In *Rance*,^28^ Brooke J suggested a fetus would be viable only when capable of ‘living through its own lungs alone, without deriving any of its living or power of living through any connection with its mother’.^29^ English law clearly focuses on the capacity to breathe to establish viability. In *C v. S*, Mr C claimed that capable of being born alive was a more restrictive legal concept than viability; it need only be established that the fetus could survive no more than birth.^30^ It was held that being born at 18 weeks would result in little hope of survival, because there would be no capacity to breathe. This did not resolve the issue of whether the capacity to breathe only for a short time after birth would be sufficient, or whether the capacity would have to be more substantial (such as longer term use of the lungs). Intuitively, capable of being born alive is intended to convey a capacity to breathe for some reasonable time after birth.^31^ The wording of the ILPA 1929 implies that the offence would be committed when a fetus is delivered alive and breathes but does not live long after that. The presumption in the AA 1967 that a fetus is capable of being born alive from 24 weeks gestation demonstrates Parliament’s intention to include fetuses that once born may not survive in the longer term, given the usual prognosis of neonates born at this point in gestation, which would have been even worse 20 years ago when the threshold was made law.^32^ Viability in English law means capable of being born alive and surviving for a time by breathing, rather than being born alive and surviving in the longer term.

### B. The USA

In *Roe v. Wade*, the Supreme Court recognized that the constitutional right to privacy encompasses the right to terminate a pregnancy, but this right could be qualified by the state’s interest in potential life at fetal viability. The right to abortion continues until the point a fetus becomes ‘potentially able to live outside the mother’s womb, albeit with artificial aid’.^33^ Blackmun J advanced a ‘trimester framework’ to separate different developmental phases in gestation and illustrate the differing levels of state interference that could be lawfully justified in each. *Roe* holds that ‘with respect to the State’s important and legitimate interest in potential life, the ‘compelling’ point is at viability… if the State is interested in protecting fetal life after viability, it may go so far as to proscribe abortion during that period, except when it is necessary to preserve the life or health of the mother’.^34^ This point was identified as the third trimester of pregnancy. Nineteen years later in *Casey*,^35^ the Supreme Court, while abandoning Blackmun’s trimester framework, reaffirmed ‘that viability marks the earliest point at which the state’s interest in fetal life is constitutionally adequate to justify a legislative ban on non-therapeutic abortions’ emphasizing that ‘the attainment of viability may continue to serve as the critical fact…’.^36^ The Court replaced the trimester framework with the ‘undue burden’ test, holding that a law is unconstitutional if its ‘purpose or effect is to place substantial obstacles in the path of a woman seeking an abortion before the fetus attains viability’.^37^

Pregnant people have the right to terminate pregnancy, as part of the constitutional right to privacy, until foetal viability. Individual states must refrain from passing laws that unduly interfere with access to abortion until viability. After viability, States can pass whatever restrictions on abortion they see fit, expect that a pregnant person must still be able to access abortion if their pregnancy poses a serious risk to their health or life. This clear delineation by viability begs the question about its definition. In *Casey*, viability was defined as ‘the time at which there is a realistic possibility of maintaining and nourishing a life outside the womb, so that the independent existence of the second life can in reason and all fairness be the object of state protection’.^38^ It was observed that the precise point of viability is dependent on medical technologies, and medical developments would come to re-determine the concept.^39^ In *Roe*, fetal viability was estimated at 28 weeks, though it was acknowledged that viability could occur earlier.^40^  *Casey* found that States could restrict abortion access from 23 to 24 weeks ‘or some moment even slightly earlier in a pregnancy’.^41^ In *Planned Parenthood v. Danforth*,^42^ the Supreme Court considered the constitutionality of a Missouri abortion statute, including its definition of viability as ‘that state of fetal development when life of the unborn child may be continued indefinitely outside the womb by natural or artificial life supportive systems’.^43^ This definition was found to be consistent with *Roe* because when a fetus is viable, it would presumably be capable of ‘meaningful life outside the mother’s womb’ flexibly understood.^44^ The Supreme Court resisted the claim that viability was a concept that could be fixed to a particular point in gestation because ‘viability was a matter of medical judgment, skill, and technical ability and [in *Roe*] we preserved the flexibility of the term…. It is not the proper function of the legislature or the courts to place viability, which essentially is a medical concept, at a specific point in the gestation period’.^45^ The viability threshold is thus seemingly dependent on technology, but ultimately on medical evidence and opinion with no discrete definition concerning the kind of independent existence of which a fetus must be capable. Viability is strongly tied to the notion of foetal independence from a pregnant person. Swyers warns that ‘assuming that medical science continues with the same momentum seen over the past two decades of advances in… postnatal care, a woman’s right to terminate her pregnancy under the current viability standard may soon disappear’.^46^

The Supreme Court’s refusal to quantify viability means that States have been left to define viability as they see fit, which has resulted in a multitude of approaches. Most State laws have ultimately followed the Supreme Court’s reasoning and maintained that viability is a medical concept, deferring judgment to medical professionals. Despite this, most States place limitations on that medical judgment by implementing some quantification of viability, either by reference to gestational age (GA) or the capacities of a fetus. Regulations at State level largely fit into three categories: those that define viability as a matter of medical judgment; those that define viability by referencing the capacities or features of the fetus; and those that define viability as a fixed point in gestation.

Some States define viability only by referencing the capacities or features of the fetus. Wyoming defines viability as ‘that stage of human development when the embryo or fetus is able to live by natural or life-supportive systems outside the womb of the mother according to appropriate medical judgment.’^47^ Utah defines viability as the point when an ‘unborn child’ is potentially able to live outside the womb as determined by a doctor to a reasonable degree of medical certainty.^48^ Among States that provide a definition of viability, the vast majority explicitly include those fetuses that can only survive *ex utero* with artificial support. As recently as 2006, Kansas defined viability as the capacity to survive after birth, ‘without the use of extraordinary measures,’^49^ placing stricter limits on the kind of *ex utero* existence considered sufficient. The law in Kansas, however, now defines ‘viable’ as the stage of fetal development when ‘it is in the physician’s judgment according to accepted obstetrical or neonatal standards of care and practice applied by physicians in the same or similar circumstances that there is a reasonable probability that the life of the child can be continued indefinitely outside the mother’s womb with natural or artificial life-supportive measures’.^50^

Interestingly, there are inconsistencies between States regarding how long after being born a fetus would have to be physically able or likely to survive to be considered viable. There are limited references to this in the legislation of most States (adopting similar language to the English model), but there are some notable exceptions. Maryland specifies that viability entails a ‘reasonable likelihood of the fetus’s sustained survival outside the womb’;^51^ Kansas also, as indicated, stipulates a ‘reasonable probability that the child can be continued indefinitely outside the womb’.^52^ Such definitions, while uncommon in State Codes, are interesting in that they, in specifying the period of time that a fetus would have to survive after birth, set a more stringent requirement on life after birth. These definitions seemingly exclude those fetuses that might be delivered alive and survive only for a few seconds of independent life.

Other States have only implicit definitions of viability. These States have statutes limiting abortion after a particular point in gestation. There is much variance on the identified point of viability from 20^53^ to 25 weeks.^54^ Some States still adopt the trimester framework, limiting abortion from the second^55^ trimester. Despite the Supreme Court having affirmed a constitutional right to abortion until viability, by having left viability abstract without careful quantification, pregnant people have been rendered vulnerable. There is extreme inequity in access to reproductive healthcare (including termination) across the Country. It is bizarre that the Supreme Court would affirm a right but leave that right with absent parameters enabling States to construct their own criteria to accessing it. There are several plausible reasons that might account for the significant discretion left to States on the content of this right. The viability threshold is broadly enunciated in law with significant scope for political interpretation.^56^

It is important to note here that in 2019 there was a trend of State legislatures attempting to abandon the viability framework entirely. A swarm of ‘foetal heartbeat laws’ has been passed in Alabama,^57^ Arkansas,^58^ Mississippi,^59^ and Louisiana.^60^ These laws attempt to prohibit abortion after a foetal heartbeat can be detected—around 6–8 weeks from conception ‘regardless of viability’^61^ (except in emergencies). These Acts have all, at the time of writing, been blocked by Federal Courts,^62^ but they demonstrate a political determination among some legislatures to challenge *Roe v. Wade.*

## IV. PRELIMINARY OBSERVATIONS

There is some legal recognition that there is a state interest in potential life in both the USA and England and Wales, which has resulted in some legislative protection for unborn fetuses. The law of each jurisdiction has also recognized that this state interest becomes ‘compelling’ later in pregnancy, so that restrictions can justifiably be enacted on abortion. There is incongruity, however, in ascertaining what viability means and the point at which viability occurs. This inconsistency demonstrates that different kinds of life *ex utero* are meaningful enough to command ‘state interest’ in each jurisdiction.

First, exactly how a viability threshold has been established in law is different in each jurisdiction. While the term viability is used explicitly in the US legal framework established in *Roe v. Wade*, it does not feature in the criminal law in England and Wales at all. Despite this, it is clear that abortion regulation constructs a gestational time limit that intersects with a fixed point in gestation that is often cited as the point of fetal viability, and the offence of child destruction affords some protections to fetuses who are ‘capable of being born alive;’ which adopts the similar logics to the concept of viability in being concerned with the fetus’s potential.

Second, there are potential differences in these jurisdictions as to whether viability as a legal concept is considered distinct from medical conceptions of viability. The US Supreme Court has been clear that viability is a term intended to reflect medical possibility, and most States have legislated along these lines, though this approach is not adopted by all State legislatures. In England and Wales, there remains debate as to whether viability is a strict legislative threshold or an ill-defined legal construction^63^ that might therefore reflect medical opinion.

Third, there is a significant inconsistency in the GA of presumed viability. In England and Wales, 24 weeks is used as ‘prima facie proof’. The US Supreme Court emphasizes that viability is a medical question, though judgments have posited fetuses which are viable from 23 weeks. There is also a significant variation in gestational limits at State level, including several US States with limits below 22 weeks. It remains to be seen how the US Supreme Court would rule on the constitutionality of these fixed points if specifically challenged. While there is inconsistency in terms of GA between and within jurisdictions, there is a common problem with all of these approaches; they all label fetuses as viable at a point in development when the data do not reflect a substantial likelihood of survival. Myrhaug and others note that since ‘proactive life support for infants born at 22–24 weeks’ GA is a relatively new phenomenon, and [therefore] we have limited knowledge on the chance of survival and survival without significant impairments’.^64^ Their systematic review concludes, however, that existing data suggest that the survival rate of neonates at 22 weeks was 7.3 per cent when calculated in proportion of all live births, and 24.1 per cent as a proportion of infants transferred to neonatal intensive care for treatment.^65^ At 24 weeks, the survival rate is around 29.9 per cent when calculated as a proportion of all live births and 59.7 per cent as a proportion of those transferred to neonatal intensive care. Even at 24 weeks, the likelihood of survival is dependent on access to the best medical care.^66^ Some cohort studies included in the review placed the likelihood of survival much lower.^67^ It is puzzling that the law, and medical profession, sets the viability standard with reference to, effectively, some chance of survival, rather than a reasonable chance of survival.

Moreover, there is no evidence that a fetus could ever survive *ex utero* at some of the points that legislatures have identified (less than 22 weeks) even with intensive care, because the lungs would not yet be formed. Survival with the aid of intensive care is dependent on a neonate having sufficiently formed lungs to tolerate artificial ventilation.^68^ The way these thresholds (at a specific GA) have been constructed suggests that viability is concerned with a chance of survival, rather than the likelihood of survival or meaningful life after birth. Intuitively, however, it seems that a ‘state interest’ in an entity would be more defensible if there were some longevity to life.

In the following sections, I explore the coherency of viability in the law, by examining what kind of life *ex utero* the legal frameworks of England and Wales and the USA consider valuable. This turns on whether viability is constructed as a rebuttable presumption or an evidential rule. In both jurisdictions, an isolated point in gestation is identified, formally or informally, as the point a fetus is (assumed) viable. Does this preclude fetuses younger than this point from being recognized as viable? Does the law allow for the recognition that an individual fetus is not viable later in gestation? A consistent and coherent account of viability, that is carefully quantified, is not provided in English or US jurisprudence.

## V. VIABILITY EARLIER IN GESTATION

In England and Wales, the law has constructed viability as a rebuttable presumption, because a fetus before the 24-week threshold is not precluded from being determined ‘viable’. The *C v. S*^69^ judgment demonstrates the willingness of English judges to examine evidence regarding the viability of fetuses before the 24-week threshold.^70^ However, the standard remains ‘capable of being born alive’, suggesting that the fetus must be capable of displaying some legally recognizable life signs after being born. A newborn capable of only surviving with the aid of intensive care is considered viable in English law. There has been limited further clarification about what likely duration of life after birth is sufficient to establish that a fetus is viable. Viability is even less fixed in US law since the Supreme Court has refused to fix a point in gestation from which a fetus is presumed viable. Individual States have thus been free to define viability and the point from which they will assume that fetuses are viable. Some states set a low threshold of presumed viability.

Anti-choice campaigns often attempt to validate arguments about reducing abortion time limits by referencing advances in medical technology and their impact on the viability timeline.^71^ The argument is that if a fetus can survive earlier in gestation *ex utero*, then abortion legislation should reflect that medical possibility. Prematurity remains the leading cause of death for preterms born at or before 26 weeks’ gestation,^72^ because before this point, the infant risks being too functionally immature to survive. The likelihood of survival, and without serious complications resulting from care complications or developmental limitation, increases with GA. There is considerable variation in studies investigating survival rates for neonates born preterm and extremely premature that is usually, as the British Association of Perinatal Medicine note, because results are influenced greatly by ‘cohort selection, place of birth and variation in the provision of active and obstetric and neonatal management’.^73^ However, a recent systematic review notes that chance of survival, among those who receive intensive treatment, increases from 24.1 per cent at 22 weeks to 90 per cent at 27 weeks.^74^ Only 23 per cent of surviving children delivered at 22 weeks are without an impairment; this rises to 39.3 per cent of those born at 24 weeks and to 70.8 per cent at 27 weeks.^75^ In the medical literature, there is generally some consensus that a realistic threshold based on some chance of survival (as explained) can be placed at 24 weeks.^76^ However, notable exceptions of preterms surviving exceptionally young and the wishes of parents to attempt resuscitation on preterm neonates serve to reinforce the notion of a viability threshold accounting for outside possibilities. The Nuffield Council of Bioethics guidelines regarding resuscitation decisions specifies that resuscitation attempts on newborns below 22 weeks should not be attempted outside of recognized clinical trials.^77^ This raises questions about the kind of life the law is recognizing *ex utero*. Is some possibility of survival *ex utero* sufficient, or some possibility of a healthy life with longer-term survival prospects? Or even a reasonable likelihood of survival with good prospects for a healthy life? In both jurisdictions, law and medical guidelines do not set their sights as high as a reasonable likelihood of survival and/or long-term prospects.

### A. Artificial wombs: artificial amnion and placenta technology (AAPT)^78^

There has been little change in the data regarding premature survival in recent years, as it appears that the clinical possibilities of conventional care to aid survival have been exhausted.^79^ Interventions can only do so much for preterms born without the functional capacities for independent life, e.g. insufficiently formed organs. There is, however, technology on the horizon thought capable of shifting the viability timeline earlier in gestation. Two research teams, in the USA and Australia/Japan, have claimed ‘proof of principle’ for artificial amnion and placenta technology^80^ (AAPT) and speculate that their devices might replace conventional neonatal intensive care in the future.^81^ These devices are designed to mimic the function of the placenta and environment of the human uterus such that they are capable of continuing the process of gestation (known as partial ectogestation^82^); ‘the central principle underlying the iterative development of [the EVE] platform is to treat extremely preterm infants as fetuses, rather than as small babies… to avoid the use of pulmonary gas exchange’.^83^ Therefore, AAPT would not be subject to the same limitations of gestational maturity, as they can facilitate continued organ maturation and growth and so could shift perceptions of the viability timeline.^84^

Research scientists are explicit that they intend to mitigate the impact of being born premature rather than challenge current conceptions of viability. They identify their clinical target population at 23–25 weeks,^85^ as those who would already be subject to treatment in intensive care. I have argued elsewhere that in the initial stages of testing this technology, it should be used on those preterms that we would not consider viable, because to test it on preterms potentially able to survive in intensive care is to deny them medical treatment for potentially no benefit.^86^ There is thus the possibility that clinical trials of AAPT will necessarily demonstrate the clinical utility of the technology in aiding developing human entities not currently considered viable. Moreover, if AAPT can better promote the survival of preterms on this current viability threshold, there will be calls to use the technology to aid those preterms delivered not far behind it.^87^ Gradual use of technology, primarily to aid individual patients and eventually informing general practice, is how the medical conception of viability has arrived at the 22–24 week point. It is hard to speculate about how far AAPT may be able to stretch the viability timeline, as there will be other natural limitations. The developing human entity must survive a form of birth to be placed in an ‘artificial womb’ (AAPT). The developing human entity must also have some kind of fetal physiology to be supported by the current models being developed by researchers in the USA, Australia/Japan, and the Netherlands.^88^ In the future, odds in the AAPT might be improved if caesarean sections are scheduled and exposure time and stress for developing entities limited. Future models may (a long way in the future) also be capable of sustaining even more primitive human entities. While AAPT is a speculative development, the technology is an interesting example to examine the coherence of viability in the law.

AAPT is conceptually distinct from other forms of preterm care because it is continuing the process of creating, rather than rescuing, developing human entities. Thus, the subject of an artificial womb (AAPT), termed the ‘gestateling’,^89^ is a unique entity because it is neither a fetus nor a neonate. A fetus is a developing human entity undergoing the process of gestation dependent upon a pregnant person. A neonate, while developing still, is no longer undergoing a process of creation and must be capable of partially self-sustaining in the external environment. A gestateling is undergoing the process of gestation and is not *ex utero* in a meaningful sense, but it is not dependent upon a pregnant person.^90^ Viability is generally thought of as the capacity to exist *ex utero* (conventionally the uterus being a part of a pregnant person). There is the potential to describe some fetuses as ‘viable’ in the sense that they can be sustained outside the body of a pregnant person, but they are not in any way capable of an independent post-gestation existence. Should we consider these fetuses ‘viable?’ Or not, because they are capable only of being transferred to an ‘artificial womb’?

Insofar as any legal concept of viability has any utility, and it would distinguish between those fetuses that are capable of independent existence after gestation and those that are not. A fetus becomes ‘viable’ only when it can survive ex-gestation irrespective of where that gestation is taking place. A fetus should only be described as ‘viable’ only when able to take on some of the burden of sustaining themselves independently. Emergence from gestation involves the developing human entity undergoing meaningful biological adaptations enabling self-sufficiency, interaction with and survival in the *ex utero* environment (even if they were dependent on rescue technologies in neonatal intensive care).^91^ There are substantive reasons to believe that a fetus gestating in a pregnant person that would only be able to continue any biological life *ex utero* if the process of gestation was continued is different in nature to the fetus capable of making the biological state changes for independent living. If the purpose of the viability timeline is to identify potential life that the state has a meaningful interest in preserving, the state has a greater interest in the preservation of a life able to survive *ex utero* rather than an entity that can only ‘continue to be gestated *ex utero*’.^92^ The entity that can make a meaningful adaptation to the external environment is a life that has the potential of being realized, rather than having potential only in a removed sense.

Is the existing law of England and Wales and the USA nuanced enough to account for this difference? Doctrinal lawyers are often criticized for considering the application of contemporary legal frameworks in different contexts—for example, the development of a future reproductive technology. However, it is only by speculating about how a potential future event (the development of AAPT or ‘artificial wombs’) might be treated in contemporary legal frameworks that we can expose any potential conceptual flaws in the existing legal framework^93^ and consider the necessary changes for a better legal future. In such an exercise, therefore, we can consider how the law and the logics that underlie it need reform in line with technological developments.

While English law does, to some extent, entrench a moveable concept of viability, it makes explicit reference to being capable of ‘being born alive’ rather than just existing *ex utero*. The gestateling (subject of AAPT) would likely not be capable of satisfying the legal definition of ‘born alive’ in English law because it does not breathe or perform any exertive activities that demonstrate any independence or self-sufficiency.^94^ Because it remains in the process of gestation, it is not ‘born alive’. Thus, fetuses should only be considered viable when capable of maintaining some kind of (supported) independent existence following the biological state changes encompassed in birth. In the USA, scholars frequently explain that the Supreme Court quantified viability as a standard of only ‘independent existence’ from a pregnant person.^95^ Horn, in her comparative account of abortion and the law in England and Wales, the USA, and Canada after the development of artificial womb technology, stresses that the viability timeline in US law does mean that the development of the technology could result in significant restrictions on abortion access.^96^ The explicit references to medical advancements and the likelihood that viability will shift with future medical developments in *Roe* and *Casey* are highlighted as support for the suggestion that a fetus would be considered viable for legal purposes earlier and earlier in gestation.^97^

English and US law thus have different approaches: English law is concerned with fetuses capable of being born and maintaining some independent life function, whereas in US law, the standard is stipulated in a way that sets the threshold lower; at just independence from a pregnant person. English law is concerned with the capacities of the developing human entity, whereas US law is not. Some US States have passed legislation conceptually similar to the English approach—making specific reference to the capacities of the fetus—even though they are not required to set such specific standards. The reasons for the difference can be elucidated in the origins of the law in each jurisdiction. In England and Wales, the focus on the ‘capacity to be born alive’ standard originates from the ‘born alive’ rule in criminal law. A human entity only has legal personality and can therefore be the victim of homicide, if it is born alive*.*^98^ Such a rule has been long established in English law; in the 17th century, Sir Edward Coke wrote that

if a woman be quick with childe and by potion or otherwise killeth it in her wombe, or if a man beat her, whereby the child dieth in her body, and she is delivered of a dead childe, this is great misprison, and no murder: but if the childe be born alive, and dieth of the potion, battery, or other cause, this is murder; for in law it is accounted a reasonable creature, in *rerum natura*, when it is born alive.^99^

A human entity that is killed before being born alive cannot be recognized as a victim of homicide, but its death is recognized in the crime of child destruction.^100^ The offence of child destruction was constructed with reference to the ‘born alive’ rule—and therefore places emphasis on the potentiality to be alive (i.e. exercising some independent function), since being alive at birth is the relevant fact in the law.^101^ In contrast, US law has focused on the capacity for an independent existence because the law as it was conceptualized was not attempting to construct a coherent distinction between infanticide and homicide, but to establish an object of legitimate state interference—thus, it focuses solely on the circumstance in which a human entity’s existence no longer infringes on the right to privacy of a pregnant person by virtue of its location—independence from the pregnant person.

It is interesting that in both jurisdictions, there remains some extent to which the determination of viability (even for the purposes of the law) is a medical question. The US Supreme Court is, as explored, explicit that this is the case, and in England and Wales, the ‘capacity to be born alive’ may be open to medical interpretation. It remains to be seen how the medical profession will respond to AAPT and whether the technology will be considered conceptually distinct from rescue technologies in practice and its impact on the viability timeline. For obvious reasons, there is yet little qualitative or quantitative data available about the attitudes of doctors toward viability and abortion provision in light of AAPT. In a 2020 study, 91 Australian doctors were asked closed questions about their conception of viability in the advent of such technology and its capacity to continue gestation of fetuses at 22 weeks. The study reported that 88 per cent of respondents believed that, in light of the technology, if a fetus were delivered at this point, it should be considered ‘viable’.^102^ Interestingly, 41 per cent of respondents indicated that the availability of such technology would influence their opinion of abortion being performed at this GA.^103^

If viability is a concept intended to meaningfully convey some notion of the potentiality of life, English law currently adopts the more coherent account of viability as applied earlier in gestation. There is a meaningful developmental difference between a fetus no longer necessarily in need of being created because it could survive after gestation with conventional care and a fetus that cannot be sustained outside of gestation. Though questionable, if the state has an interest in potential life, it seems intuitive that this interest would be directed only toward those fetuses that could live in the external environment rather than those human entities still dependent on being created (whether *in utero* or an ‘artificial womb’). The approach of the US Supreme Court is vague and encompassing of fetuses incapable of making the necessary biological state changes to demonstrate a completed birth**.**

## VI. VIABILITY LATER IN GESTATION

The legal framework of these jurisdictions considers viability a rebuttable presumption earlier in gestation, but what about those cases where the viability of a fetus later in gestation (passed the point the law might presumes viability) is questionable? Examining to what extent the law is sensitive to the capacities of an individual fetus is an important aspect of ascertaining the substance of viability and what it intends to protect. If viability is about establishing the point a fetus becomes a ‘potential life’, this will always be specific to an individual fetus and its capacities. If viability operates as a rebuttable presumption in one direction, to allow for recognition that a fetus earlier in gestation might be viable, it would be inconsistent not to recognize that some fetuses may not be viable later in gestation. It is potentially possible to generalize that most fetuses will be sufficiently developed to survive with some assistance after a specific point in gestation; however, this will always depend on the particular circumstances. The medical standard of viability is based on the ‘human interpretation of statistical probabilities’ applied to fetuses as a class.^104^ There will be variances between fetuses at the same point in the gestational period, such that we could describe one fetus as ‘viable’ at 25 weeks and another not based on their stage of development.

### A. Anencephaly

Anencephalic fetuses have a congenital absence of the brain, parts of the skull and scalp,^105^ and are inherently non-viable because the absence of a brain is lethal and irreversible in all cases.^106^ Stillbirth is a common outcome of an anencephalic pregnancy.^107^ When the fetus can be delivered biologically alive, it does not survive long post-delivery.^108^ Anencephaly is usually detected during prenatal ultrasounds; though sometimes not until later in pregnancy.^109^ Applying the law to anencephaly allows consideration of whether viability is individualized to a fetus and its capacities, or it is a rigid concept strictly applied later in gestation.

In English law, there is provision for the termination of an anencephalic fetus even after 24 weeks (the implicit viability threshold). The Abortion Act 1967^110^ provides a defence to a doctor performing a later-term abortion in the case of ‘a substantial risk that if the child were born… it would be seriously handicapped’. There has been little clarification of the necessary severity of the handicap in these circumstances;^111^ however, it is clear that given the severity of anencephaly that abortion would be lawful here. This does not answer the question as to whether such a fetus would be considered ‘viable’ and whether the prima facie assumption of viability from 24 weeks can be rebutted. An English Court has not addressed this issue, because the AA 1967 does allow termination after 24 weeks in all the circumstances it is usually sought after on the grounds that the foetus is non-viable (without needing to consider viability). The AA 1967 is clear that even after a fetus is prima facie viable, the pregnant person’s interests in health and life remain paramount, allowing them to terminate their pregnancy if necessary. The handicap provision does not mandate that the fetus must have an abnormality that would be life-limiting after gestation.^112^ It is therefore unknown whether there is any requirement in English law that to be capable of being born alive, the fetus need be capable of longer-term survival. English legislation was likely intended to afford legal protection to any human entity that survived birth, irrespective of prospects for longer term survival.^113^ Viability is not a coherent concept if it does not proscribe more than surviving birth. *C v. S* established that a prima facie assumption of viability at a particular point in gestation is intended to shift the burden of proof in establishing viability away from a prosecutor. If the Court is willing to accept that a party can claim that viability is evident earlier in gestation if compelling evidence is presented,^114^ equally then should not the ‘prima facie proof’ of viability be challengeable with evidence? An anencephalic fetus, regardless of GA, would patently never be capable of surviving for a meaningful amount of time after birth. Most fetuses with anencephaly are absent a cerebral cortex and thus will never be capable of consciousness, and only capable of sustaining very limited signs of life for a short period.^115^ While such a fetus might be considered biologically alive on delivery because of these basic signs of life, it might also satisfy the legal definition of death in English law, that is brain death.^116^ Thus, describing it as ‘capable of being born alive’ seems counter-intuitive. If a fetus were never likely to have an independent life or consciousness—why should it be afforded comparable protection to fetuses with potentiality? Arguably, the AA 1967 is intended to ensure exactly this by providing explicit grounds for the termination of those fetuses unlikely to survive as a result of a severe congenital impairment.

The US Supreme Court is clear that the viability threshold intends to protect the ‘potential for independent life’. Little further clarification has been offered about what kind of life meets this standard. Thus, it remains unclear whether viability later in gestation is a rebuttable presumption in US law. Some States have chosen to be more specific in legislation about what kind of *ex utero* existence is sufficient to establish viability. While most refer to artificial life support to denote fetuses that can survive with the assistance of intensive care as potentially viable, few qualify that further with reference to the duration of life after birth. In Florida, viability means ‘the stage of fetal development when the life of the fetus is sustainable outside the womb through standard medical measures’.^117^ In Maine, viability is defined as ‘the state of fetal development when the life of the fetus may be continued indefinitely outside the womb by natural or artificial life supportive means’.^118^ Maryland defines viability as a ‘reasonable likelihood of the fetus’s sustained survival outside the womb’. The American College of Obstetricians and Gynecologists echo this approach, advising that abortions performed later in pregnancy are usually those involving risk to the pregnant woman or foetal ‘anomalies incompatible with life, such as anencephaly…’.^119^ In some States, a fetus would be described as viable if there were only the possibility of it surviving birth and no longer, for example States that consider a fetus viable from a specific point in gestation at which the data would not predict a reasonable likelihood of either long-term survival or even any survival after birth.^120^ The recognition afforded to these fetuses with conditions incompatible with life is not only incoherent but also actively harmful to pregnant people carrying an anencephalic pregnancy. A recent anonymous testimonial by an American physician describes the agony of attempting to support patients carrying pregnancies with no chance it results in a child while being legally, professionally, and politically restricted from offering the option of ending the pregnancy.^121^ The legality of an abortion based on foetal abnormality is dependent upon the law of individual States.

The rigidity of homicide law in both jurisdictions means that the live birth of a non-viable neonate that is subsequently killed would trigger homicide culpability. In the USA, the Born-Alive Infants Protection Act 2002 extended federal protection afforded to infants ‘born alive’ at any stage of development. The Act defines ‘born alive’ as breathing or ‘has a beating heart, pulsation of the umbilical cord, or definite movement of voluntary muscles…. Regardless of whether the expulsion… occurs as a result of natural or induced labor, cesarean section, or induced abortion’.^122^ It is possible for a fetus without the capacity for life; even those absent a brain, to be born bearing these signs of life, and protected by the law of homicide.

The ‘born alive’ rule also applies in England and Wales. Any human entity born alive is a person for the law of homicide. English law dictates that a child is born alive if it breathes or demonstrates ‘any other signs of life’.^123^ There was little further clarification in the relevant legislation about what signs of life are sufficient to demonstrate life. It is clear, however, that a fetus need only survive the process of birth and display signs of life for a short time after to be a potential victim of homicide if action is taken to end its life.^124^ In both jurisdictions, the live birth of a non-viable neonate that is then killed would trigger homicide culpability, yet termination of the same entity when a non-viable fetus (whether it is non-viable inherently because it is absent a brain or because it is too immature to survive) might not be an unlawful abortion. This appears initially incoherent and confusing and is an inconsistency that anti-choice advocates often attempt to monopolise on by analogizing the unborn with the neonate. In the USA, this has culminated in several States adopting ‘foetal personhood’ laws to recognize that a fetus can be the victim of a homicide with the effect of undermining abortion provision.^125^ There has been no positive case advanced for non-viable or never-viable fetuses being afforded the protection of homicide law. It is illogical to use a fetus that was never capable of living long post-birth to reason that a fetus also not capable of, or even never capable of, living if it were born should have an equivalent protection. This was the conclusion reached on this subject in the Supreme Court of Brazil which held that, ‘abortion is a crime against life. The potentiality of life is what is protected. In the case of anencephalic fetuses.... there is no life possible’ thus, there can be no crime’.^126^ In any event, we expose more incoherence in the meaning afforded to viability in the law when we consider examples later in gestation, both within and between the USA and England and Wales.

It is useful here to highlight that there is no moral continuity between an entity that has actually been delivered alive and an entity that could be delivered alive.^127^ The rigidity of homicide law in this area is potentially necessary as a way of ensuring that there is no discrimination between human entities. Greasley explains that ‘interpersonal variations in person-relevant capacities should not affect the equal moral status of individuals within the range… [this is] morally essential for maintaining the kind of relations between persons that we deem valuable’.^128^ As such, a human entity delivered alive^129^ is afforded the protection of the law of homicide because they have been born alive, even if they cannot survive in the longer term. It is clear, however, that a fetus who has the capacity to be born alive (whether that survival would be short- or long-term) need not be treated with a comparable level of respect. The reality is that capacity to be born alive is not the same as having been born alive.

Any recognition given to the ‘capacity to be born alive’ is an argument based on potential—and we do not treat entities on the basis of what they might be, but on what they are.^130^ Any equivalence drawn between the capacity to be born and having been born is ‘in itself misleading, for it is often taken to suggest that an X that is potentially a Y in some mysterious fashion already possesses the being and significance of Y’.^131^ There are meaningful differences between these two entities—one capable of being born alive and one born alive—most notably, that the entity capable of being born alive is still located inside a gestating person,^132^ or dependent on an artificial placenta. In contrast, however, the entity born alive is ‘natal’ and has ‘come into the world with and as a specific body, in a given place, set of relationships, situation in society, culture, and history…’ and is dependent in ways that specifically flow out of their birth^133^ including interaction and physical contact with others. Entities with the potential to be born alive may well become natal in this meaningful sense, however, significant changes in physicality (the process of birth), in the case of entities located inside a gestating person, and physiology (adaptation to the external environment) must take place before they do. These are substantial prerequisites to being born alive: a human entity must survive delivery and must adapt to the external environment.

## VII. THE CONCEPTUAL ILLEGITIMACY OF VIABILITY THRESHOLDS

Horn argues that ‘Canadian law does not set a viability timeline for abortion demonstrates that legal “viability” is not a quality naturally vested in some fetuses, but a fictive construct designed to act as a limitation on abortion’.^134^ She makes this argument as a basis for decriminalization as a model for protecting abortion provision with the advent of artificial womb technology in the UK (where the AA 1967 applies) and the USA like, as she demonstrates, the right to abortion would be in Canadian law even with such technology available.^135^ The traditional viability analysis, evident in the law in English and US law, assumes that foetal viability is a legitimate foundation for regulation. Horn concludes that a framework ‘based on fetal viability regardless of how it is defined fails to sufficiently account for relationality and care for the pregnant person’ by decentralizing the pregnant person.^136^ In this paper, I sought to add meaningfully to her call—first, by demonstrating the doctrinal incoherence in the concept of viability as it both currently quantified and deployed in English and US law. This investigation was necessary to illustrate how the ‘self-evident logic’ that is sometimes claimed of the viability threshold does not stand up, especially if we examine how viability is defined and utilized in the law. Second, my work adds to this call by demonstrating the inadequacy of viability in regulating abortion provision in contexts beyond that of AAPT; most notably, by comparing how the concept applies to fetuses with conditions incompatible with life. In the following section, I make the case that using a viability threshold to regulate abortion provision is not an effective standard of compromise in the law, nor conceptually legitimate.

The entrenched reference to viability in the law assumes that it is necessary to strike a balance between a state’s interest in potential life and a woman’s right to bodily autonomy and equality. Frequently, the viability threshold is conceptualized as a ‘compromise’ between these two interests, but it performs no such function. Despite the regulation of abortion by criminal statute in England and Wales and the USA, it is notable that the state interest in abortion policy has not been elaborated upon in any of these jurisdictions. Dalzell explains that in *Roe* and *Casey*, there is no exploration of the ‘state interest in potential life’ by quantifying or defining this interest, effectively ‘stripping the state from the duty of proving its interest’.^137^ The interest in potential life has been interpreted broadly in the USA, as always present but ‘compelling’ only after viability in *Roe*,^138^ and in England and Wales, there has been little exploration of the justification for state rationale in legislating to make abortion harder to access after a viability threshold. Abortion legislation was introduced in England and Wales ‘to clarify the law for doctors and to stem the misery and injury from unhygienic, risky illegal abortions’.^139^ In the 1990, Commons debate regarding the time limit for ‘social abortion’ discussion centered around preventing later-term abortion, and a lack of public support for abortion ‘on demand’^140^ rather than explaining why abortion was a public, rather than a private, matter. What is the justification for abortion being anything other than a person’s private choice? All sorts of ‘moral matters’ that were at one time thought the business of the state have since been rightfully recognized as private choices. The onus in thinking about abortion must be flipped; claims that the state has an interest in preventing abortions must be fully justified, rather than it being women and pregnant people who are routinely forced to defend their privacy, autonomy, and equality.

If the justification for state interest is based on preserving life, or potential life, in any real sense, the law would only provide legal protection for a developing human entity from the point of birth. This is the point at which there is a human being existing independently of another person demonstrating any potentiality for independent life. This is just not true in any way at the point at which an unborn entity is dependent upon a pregnant person. There is compelling evidence to suggest that only fetuses near to or at the end of the gestational period can be described as ‘naturally viable’.^141^ The gestational period is around 36 weeks, and a fetus is only likely to have sufficiently developed lungs, allowing for breathing *ex utero* without assistance, at around 30 weeks.^142^ There might be an attempt to argue that the State has an interest in life in a symbolic sense—in setting a standard of protecting life that might come to fruition as a way of recognizing the value of life. This cannot be the case, however, as a State that professed such a position would surely pass rules limiting the use of birth control, or requiring that ‘spare’ embryos left over from IVF be implanted, as these examples are instances where there is interference with ‘life’ in a symbolic sense.

The balance in the ‘viability compromise’ is not appropriate because it pits women and pregnant people’s rights against unarticulated state interests. As Jackson highlights ‘the logical corollary of an abortion law that sets limits upon women’s access to abortion is that, in certain circumstances, a woman can be obliged to carry her unwanted pregnancy to term’.^143^ The purpose of a viability threshold limiting abortion access, therefore, will always be to place some (symbolic or real) limits on pregnant people’s rights. The argument could be made that, even if AAPT were available tomorrow, a shift in the viability threshold is unlikely to be so drastic to restrict access to conventional abortion. The vast majority of abortions take place before 13 weeks in England and Wales^144^ and the USA.^145^ Thus, most pregnant people would not be prevented from accessing abortion. However, as Eades explains those who face difficulty are likely to be the most vulnerable—pregnant people who experienced delayed seeking abortion because they live in an area where access is difficult, they have experienced domestic violence or are younger or older persons who did not recognize their symptoms as pregnancy.^146^ These people are equally entitled to access healthcare and should not have to face the stigmatizing effects of being labeled as an ‘exception to the rule’ for having a post-‘viability’ abortion. Even if few pregnant people are denied an abortion, the impact of singling out more vulnerable people in difficult situations for the stigmatizing process of proving the necessity of their abortion, or face losing the option, is substantial.

Regulating abortion by reference to a viability threshold is not a compromise because it fails to recognize the importance of the right to meaningful equality for women, penalizes pregnant people likely to submit for abortion later because of circumstances that have already marginalized them, and labels women and pregnant people as ‘in need of regulation’ to prevent them making ‘bad’ choices. Cook explains that

criminal abortion, like crime generally, is a legal and social construct … the criminal essence of abortion then implicates the social construction of those who actually and potentially seek abortion and those who provide and assist in its provision. By framing abortion as a crime societies ascribe deviance to those seeking and providing it….^147^

The framing of all abortion as a crime in England and Wales ‘in need of a medical explanation’ has a similar effect of stigmatizing the choice to end a pregnancy^148^ and constructing those pregnant people who access treatment as deviant and as failing to perform the role expected of the female body. This will necessarily affect how doctors perceive the procedure, those seeking it and the advice that they give. This is inappropriate because it limits access to a service that is necessary both to ensure female health and to ensure that women have access to social equality. Cornell argues that there can be no meaningful equality for female people without access to abortion because denying access ‘prevents the minimum conditions of individuation necessary for any meaningful concept of selfhood’.^149^ She posits that denying access to abortion ‘should be understood as a serious symbolic assault on a woman’s self of self precisely because it thwarts the projection of bodily integration and places the woman’s body in the hands and imagings of others who would deny her coherence by separating her womb from herself’.^150^ This harm is broad, experienced not only by female people at the time of unwanted pregnancy but also constantly by all people with the physiology to get pregnant. Without abortion, all people with the physiology to get pregnant are denied that their womb (and their body) is theirs to imagine into the future. Cornell explains that ‘the fear of unwanted pregnancies and illegal abortions haunted women’s sense of themselves long before the women themselves actually became pregnant’ and thus to deny a right to abortion is to label the female body as not individual (and its function to be subjectively determined), but as limited to a maternal function.^151^ A person cannot perceive themselves as equal to others in the course of social life if they are not able to engage in social activity without feeling defined by their sex or physiology, thus limiting their free choices (particularly with regards to sexual activity). Interference in choices about abortion in the law, which are also based on archaic assumptions about pregnancy and female behavior, attempts to enforce notions of the female body as inevitably constrained to the maternal function by biology and thus reinforces problematic notions of female function. Viability is not a compromise at all.

There are also pragmatic problems with using a viability threshold to regulate because it is innately arbitrary. Viability is wholly dependent on geography and resources.^152^ Moreover, it is moveable and uncertain.^153^ The standard of viability in medicine is based on the ‘human interpretation of statistical probabilities’ applied to fetuses as a class.^154^ There is disagreement about the likelihood of survival at different points in the gestational period. There is, for example, significant dissenting medical opinion about the appropriate point to rule out ‘newborn rescue’ after birth.^155^ There is also variance between individual fetuses at the same point in the gestational period. The US Supreme Court, in both *Casey*^156^ and *Roe,*^157^ explained that legislatures and Courts are entitled to draw arbitrary lines so long as they produce fair outcomes. Limiting abortion access based on viability always perpetuates unfairness, because it forces clinicians to engage with the concept of viability and, as Horn explains, thus think more about GA and the fetus than the pregnant person and their needs.^158^ Thus, again the focus on viability is not an ‘effective compromise’ between abortion rights and state interest in fetal life. It might be, however, argued that such considerations are relevant from the perspective of the pregnant persons’ health because there are material risks associated with intervention later in gestation. Erdman demonstrates, however, that abortion is

often targeted for excessive regulation due to falsehoods about its inherent risks or dangerousness, [which is simply] a function of abortion stigma. The over-regulation of abortion throughout pregnancy on grounds of medical need or safety is another instance of boundary crossing, where moral and material hazards merge.^159^

Restrictions based on these grounds, Erdman explains, overstay the available evidence and thus prevent the safe delivery of healthcare services to pregnant people.^160^ The construction of ‘absolute gestational cutoffs’^161^ prevents the (what should be undisrupted) human rights of the pregnant person to make decisions about their own healthcare.^162^ This right of pregnant persons is well recognized in the law,^163^ and yet seems to go completely ignored in this context. Moreover, the arbitrariness of viability in some legal instruments constructs an imprecise gestational cut off, which even further harms pregnant persons, by actively fostering moral uncertainty among providers that has come, and will continue, to limit pregnant people’s access to care from their doctors.^164^

Furthermore, because viability is innately variable, it has been utilized by the anti-choice lobby and US State legislatures to justify restrictive definitions only loosely related to the capacity to be born alive. ‘Foetal pain’ has been related to viability in attempts to embed further restrictions in federal law.^165^ It is hard to see why the state has an interest in a fetus just because it has a primitive ability to feel pain (rather than the complex emotions associated with pain only experienced when one has the ability to subjectively interpret experiences such that pain becomes suffering). Or, why the state has an interest in a fetus that can be gestated *ex utero*, or even survive *ex utero*, when this entity is not a fully formed human being and, at the time of making these assertions of interest, is located inside a person. The viability timeline is primarily useful for aiding doctors and parent(s) in making decisions about the intensive care that should be provided to preterm neonates based on their functional capacities to survive in the external environment. This is an entirely different situation from people making decisions about pregnancy termination—so why should the two influence each other?^166^ The viability threshold assumes that legislation is necessary to prevent a surge in late-term abortions when this is unlikely. The data in countries where abortion law makes no reference to a viability threshold suggest, however, that there has been no quantum leap in abortion (or late-term abortion) provision in the absence of law.^167^ Pregnant people seeking abortion, for a variety of reasons, tend to access treatment as early as possible. Thus, without explaining what the state interest is in the viability threshold, it becomes easy to presume that it is intended to do nothing other than control both literally and symbolically the female body, women and pregnant people’s choices.

Jackson advocates that English ‘abortion law should no longer consist in a set of defences to the crime of abortion, but rather should, like the rest of British medical law, be informed by the guiding principle of self-determination’.^168^ If abortion is recognized in England and Wales as a healthcare resource, meaningful conversations can be had to protect access to preserve pregnant people’s bodily integrity, healthcare interests, and women’s equality and, agreeing with Horn, even if medical technology in neonatal intensive care advanced to the point of AAPT.

The AA 1967 does not apply in Northern Ireland, which meant that (until recently) abortion under the Offences against the Person Act 1861 was criminal except where necessary to save the pregnant person’s life. In 2019, abortion was finally decriminalized in Northern Ireland.^169^ This step signaled progress in the UK and an opportunity to consider how abortion might be regulated without over-medicalization or unnecessary gestational time limits. However, on the March 31, 2020, the Abortion (Northern Ireland) Regulations 2020 came into effect^170^ and this new regulatory framework still entrenches abortion provision by reference to gestational limits. Regulation 3 stipulates that a medical professional can terminate a pregnancy where they are of the opinion, formed in good faith, that the pregnancy has not exceeded its 12th week. This provision is more progressive than section 1 of the Abortion Act 1967 because it does not require that an abortion before 12 weeks be justified in clinical terms. An abortion can be performed for any reason or no reason at all. However, that this is limited to 12 weeks is unfortunate. Regulation 4 effectively mirrors s.1 (1) (a) of the AA 1967 and stipulates that where a pregnancy has not exceeded 24 weeks termination may be provided where the continuance of pregnancy involves risk to the physical or mental health of the pregnant person greater than termination.^171^ After this point, pregnancy may only be terminated where there is some immediate necessity to save the life of the pregnant person,^172^ where there is risk to life or grave permanent injury of the pregnant person^173^ or in cases of severe fetal impairment.^174^ These regulations are to be welcomed as a step toward finally enabling the provision of essential care in Northern Ireland; however, it is unfortunate that the framework still entrenches the provision of care based on gestational limits.

The advocated shift in abortion law away from rights contingent on viability advocated by Jackson,^175^ Sheldon,^176^ Erdman,^177^ Horn^178^ and others, and the policy that would follow, might be more difficult to achieve in the USA since abortion politics are far more visceral. Thomson-Philbrook explains that abortion has become such a prominent political issue in the USA, simply because it was declared a right to be accessed by women.^179^ This illustrates an important point about how the substance of the law is in how it is socially implemented. It seems less important to have a formally declared constitutional right to services than it does to have access to them. It is beyond the scope of this paper to postulate about how a shift in abortion politics toward recognition of abortion as routine healthcare in the USA might be achieved without emboldening anti-choice strategy and jeopardizing access. Some States are already thinking about this; in 2019, Vermont passed a law guaranteeing the right to abortion with no restrictions based on timing or GA. The law is explicit that no public authority can ‘deprive a consenting individual of the choice of terminating the individual’s pregnancy; interfere with or restrict… the choice of a consenting individual to terminate the individual’s pregnancy [or] prohibit a health care provider… from terminating or assisting in the termination of a patient’s pregnancy’.^180^ The law makes no reference to GA.

## VIII. CONCLUSION

Viability is an incoherent legal concept as it is currently featured in US law and in the legal framework in England and Wales. Viability is frequently ill-defined and presumed from a point in gestation before the fetus has a reasonable chance of survival, thus undermining the (limited) logic behind the use of viability in determining the protection afforded to unborn entities. Furthermore, viability appears to be a rebuttable presumption based on the capacities of a fetus and the technology that might be available to support it in one direction (earlier in gestation), but not in the other (later in gestation) even when the fetus is completely non-viable. I demonstrated that there is, therefore, little clarity or consistency in determining what kind of life *extra uterum* matters using the examples of AAPT (‘artificial womb’ technology) and unborn human entities with life-limiting conditions. There remain significant ambiguities in the law in England and Wales and the USA because of a failure to quantify what kind of *extra uterum* existence is meaningful. In this paper, following other feminist scholars, I argued that viability was a conceptually illegitimate basis for abortion regulation.

## CONFLICT OF INTEREST STATEMENT

None to declare.

## FUNDING

I am the recipient of a Wellcome Trust Doctoral Studentship in Society and Ethics (grant reference 208245/Z/17/Z).

## Footnotes

1 J. Glover, Causing Death and Saving Lives, 124 (Penguin, 1990).

2 E.g. Joanna Erdman, *Theorizing Time in Abortion Law and Human Rights,* 19 Health Hum. Rights 31, 35 (2017).

3 D. Halfmann, Doctors and Demonstrators: How Political Institutions Shape Abortion Law in the United States, Britain and Canada 4 (Chicago University Press, 2011).

4
*Roe v. Wade,* U.S. 113 (1973).

5
*Planned Parenthood v. Casey,* 112 U.S. 2791 (1992).

6 Abortion Act 1967, s.1 (1) (a) as amended by s.37 Human Fertilisation and Embryology Act 1990, s.37.

7 The author notes that the choice of jurisdiction is also the result of their familiarity with English law and the prominence of US conceptions of viability in the discourse on abortion law. This work was completed during a research visit at the University of Toronto.

8 For a thorough comparative account of how the invention of artificial womb technology (both capable of partially or completely capable of facilitating gestation *extra uterum*) would affect both the legality of and access to abortion in the UK, the USA, and Canada see: C. Horn, Gestation Beyond Mother/Machine: Legal Frameworks for Artificial Wombs, Abortion and Care (2020) (PhD Thesis, Birkbeck University of London). This thesis more thoroughly addresses concerns related to the regulation of artificial wombs in these jurisdictions. The concern of this paper is not the regulation of artificial womb technology, but the coherency of viability as a legal construct and artificial womb technology is utilized as an example to illustrate some of the problems with the concept of viability.

9 For a full account of the earliest medical theories of fetal growth (and concepts of ensoulment) see: Véronique Dasen, *Becoming Human: From the Embryo to the Newborn Child*, in The Oxford Handbook of Childhood and Education in the Classical World 18 (Judith Evans Grubbs and Tim Parkin eds., 2013).

10 Aristotle, De Anima (On The Soul) (Penguin Books, 1986).

11 T. Aquinas, On Human Nature (Hackett Publishing, 1999).

12 William Silverman, *Incubator-Baby Side Shows*, 64 Pediatrics 127, 129 (1979).

13 Shai Lavi, *Beyond Natural Potentiality: Brain Death Pregnancy, Viable Fetuses, and Pre-Implanted Embryos,* 11 LEHR 161, 182 (2017).

14 E.g. Nuffield Council on Bioethics, *Critical Care Decisions in Fetal and Neonatal Medicine: Ethical Issues* (2006), http://nuffieldbioethics.org/wp-content/uploads/2014/07/CCD-web-version-22-June-07-updated.pdf (accessed July 27, 2019).

15 Lavi, *supra* note 13, at 182.

16
*Id.*

17 Andrew Grubb, *Abortion Law in England: The Medicilization of a Crime*, 18 Law, Med. Healthcare, 146, 149 (1990).

18 Infant Life Preservation Act 1929, s.1 (1).

19 Abortion Act 1967, s.1 (1) as amended by Human Fertilisation and Embryology Act 1990, s.37.

20 E. Jackson, Regulating Reproduction: Law, Technology and Autonomy 80 (Hart Publishing, 2011). While it may be true that the law only imposes stricter restrictions on access to abortion after 24 weeks, it is difficult, in reality, for people in the UK to access abortion after 16–18 weeks. This is because there are relatively few providers that offer late second trimester abortions. I am grateful to an anonymous reviewer for raising this point.

21 Andrew Grubb, *The New Law of Abortion: Clarification or Ambiguity?* Crim LR 659, 661 (1991).

22 Abortion Act 1967, s.1 (1) (b)–(d) as amended by s.37 Human Fertilisation and Embryology Act 1990, s.37.

23
*Id.,* s.1 (1) (a).

24 Human Fertilisation and Embryology Act 1990, s.37.

25
*C v. S,* 1 All ER 1230 (1987).

26 Grubb, *supra* note 17, at 150.

27
*C v. S, supra* note 25, at 151 per Donaldson MR.

28
*Rance and Another v. Mid-Downs Health Authority and Another* [1991] 1 QB 587.

29
*Id.* at 620–621 per Brooke J.

30
*C v. S, supra* note 25.

31 J. Mason, The Troubled Pregnancy: Legal Rights and Wrongs in Reproduction, 21 (Cambridge University Press, 2007); Abortion Act 1967, s.1 (1) (a) as amended by Human and Fertilisation Embryology Act 1990, s.37.

32 Elizabeth Chloe Romanis, *Challenging the ‘Born Alive’ Threshold: Fetal Surgery, Artificial Wombs, and the English Approach to Legal Personhood,* 28 Med. Law Rev. 99, 110 (2020).

33 Roe, *supra* note 4, at 160 per Blackmun J.

34
*Id.* at 163–164.

35 Casey, *supra* note 5, at 860 per O’Connor J., Kennedy J. and Souter J.

36
*Id.*

37
*Id.* at 878.

38
*Id.* at 870.

39
*Id.*

40 Roe, *supra* note 4.

41 Casey, *supra* note 5, at 861 per O’Connor J., Kennedy J. and Souter J.

42
*Planned Parenthood of Central Missouri v. Danforth,* 428 U.S. 52 (1976).

43 The 1969 Missouri Statute, § 2(2).

44 Danforth, *supra* note 32, at 63 per Blackmun J.

45
*Id.* at 64.

46 Matthew Swyers, *Abortion and its Viability Standard: The Woman’s Diminishing Right to Choose,* 8 Geo. Mason U. Civ. R. L. J. 87, 104 (1997).

47 WY Stat, 2019, § 35-6-101.

48 Utah Code, 2019, § 76-7-301.5.

49 Kan. Stat., 2006, § 65-6701.

50 Kan. Stat., 2018, § 65-6701.

51 MD Code Health, 2018, § 20-207 [emphasis added].

52
*Supra* note 50 [emphasis added].

53 A significant number of states have a 20-week threshold, e.g. North Carolina, N. C. Gen. Stat., 2019, § 14-45.1.

54 Virginia did have a 28-week limit, but this was revised to 25 weeks in May 2019.

55 E.g. South Carolina; C Code, 2019, § 44-41-10.

56 I am grateful to one of the anonymous reviewers for raising this point.

57 Alabama Human Life Protection Act 2019, s.2 (c).

58 Arkansas SB 134.

59 Mississippi SB 2116.

60 Louisiana SB 184

61 Mississippi SB 2116.

62 The Louisiana law has not been blocked directly, but it contained an express provision that it would only take effect if Mississippi’s law was upheld in federal courts.

63 Romanis, *supra* note 32, at 115–116.

64 Hilde Myrhaug et al., *Survival and Impairment of Extremely Premature Infants: A Meta-analysis,* 143 Pediatrics e20180933 (2019).

65
*Id.*

66 Hannah Glass et al., *Outcomes for Extremely Premature Infants,* 120 Anesth. Analg. 1337 (2015).

67 Myrhaug et al., *supra* note 64.

68 Emily Partridge and Alan Flake, *The Artificial Womb*, in Fetal Therapy: Scientific Basis and Critical Appraisal of Clinical Benefits, 83 (M. Kilby et al. eds., 2020).

69
*C v. S, supra* note 25.

70 Romanis, *supra* note 32, at 115.

71 Sheila Mclean, *Abortion Law: is Consensual Reform Possible?* 17 J. Law Soc. 106, 113 (1990); Leah Eades, *Social Realities, Biological Realities: The 24-week Fetus in Contemporary English Abortion Activism,* 74 Women’s Studies International Forum 20 (2019).

72 Kishwar Azad and Jiji Matthews, *Preventing Newborn Deaths due to Prematurity,* 36 Best Pract. Res. Clin. Obstet. Gynaecol. 131, 132 (2016).

73 British Association of Perinatal Medicine, Perinatal Management of Extreme Preterm Birth before 27 weeks of gestation, https://hubble-live-assets.s3.amazonaws.com/bapm/attachment/file/182/Extreme_Preterm_28-11-19_FINAL.pdf (accessed Apr. 21, 2020), 18.

74 Myrhaug et al., *supra* note 64.

75
*Id.*

76 Glass et al., *supra* note 66, at 1338.

77 Nuffield Council on Bioethics, *supra* note 14, at para 8.13.

78 Kingma and Finn explain that the term artificial womb is misleading because the womb is not ‘replaced’ by these devices—rather they are an artificial amnion and placenta. See: Elselijn Kingma and Suki Finn, *Neonatal Incubator or Artificial Womb? Distinguishing Ectogestation and Ectogenesis using the Metaphysics of Pregnancy*, 34 Bioethics 254 (2020).

79 Jennifer Hendricks, *Not of Woman Born: A Scientific Fantasy*, Case W. Res. L. Rev. 399 (2011); Elizabeth Chloe Romanis, *Artificial Womb Technology and the Frontiers of Human Reproduction: Conceptual Differences and Potential Implications*, 44 J. Med. Ethics 751 (2018).

80
*Supra* note 78.

81 Emily Partridge et al., *An Extra-uterine System to Physiologically Support the Extreme Premature Lamb,* 8 Nat. Commun. 15112 (2017); Harou Usuda et al., *Successful Use of an Artificial Placenta to Support Extremely Preterm Ovine Fetuses at the Border of Viability,* 221 Am. J. Obstet. Gynecol. 69.e1 (2019).

82 The term partial ectogenesis has been more commonly used in the literature. However, Kingma and Finn explain that ectogestation is a more accurate descriptor for the process of gestation *ex utero*. See Kingma and Finn, *supra* note 78.

83 Usuda et al., *supra* note 81.

84 These studies demonstrate that continued gestation with no complications is at least in theory possible, though they have thus far only tested their prototype AW on lambs. These studies therefore have obvious limitations. Researchers believe that their results are sufficient, however, to justify experimental application in humans in the near future. Partridge et al., *supra* note 81, at 11.

85 Partridge et al., *supra* note 81.

86
*Id.*

87 Romanis, *supra* note 79, at 752.

88 In late 2019, a third research team based in the Netherlands announced that they had received a grant to begin working on developing their artificial womb prototype. See Nicola Davies, *Artificial Womb: Dutch Researchers given €2.9m to Develop Prototype*, https://www.theguardian.com/society/2019/oct/08/artificial-womb-dutch-researchers-given-29m-to-develop-prototype (accessed Apr. 21, 2020).

89 Romanis, *supra* note 79, at 752.

90 For further defence of the conceptual distinction between the fetus, neonate, and gestateling see: Romanis, *supra* note 79; Elizabeth Chloe Romanis, *Artificial Womb Technology and the Significance of Birth: Why Gestatelings are not Newborns (or Fetuses)*, 45 J. Med. Ethics 728 (2019); Romanis, *supra* note 32; Elselijn Kingma and Suki Finn, *supra* note 78.

91 K. Greasley, Arguments About Abortion: Personhood, Morality and the Law 191 (Oxford University Press, 2017).

92 Swyers, *supra* note 46, at 105.

93 Claire Horn and Elizabeth Chloe Romanis, *Establishing Boundaries for Speculation about Artificial Wombs, Ectogenesis, Gender and the Gestating Body,* in A Jurisprudence of the Body (C. Dietz et al. eds., 2020).

94 Romanis, *supra* note 32, at 112.

95 Hyun Son, *Artificial Wombs, Frozen Embryos and Abortion: Reconciling Viability’s Doctrinal Ambiguity,* 14 UCLA Women’s L. J. 213, 214 (2005).

96 Horn, *supra* note 8, also emphasises that the development of artificial wombs is likely to impact on the availability of abortion in ways more nuanced that the shifting of the viability threshold with the development of artificial womb technology as she explains that ‘at both the federal level the ‘undue burden’ standard could allow for significant infringement on the privacy right applied in abortion both before and after viability…. Even if the ectogenic fetus was not considered viable, the state may be able to ban abortion procedures that resulted in the death of the fetus (allowing extraction to an artificial womb instead) without this being considered an undue burden on the privacy right’ at 64.

97 Robert Favole, *Artificial Gestation: New Meaning for the Right to Terminate Pregnancy,* 21 Ariz. L. Rev. 755, 764 (1979); I. Glenn Cohen, *Artificial Wombs and Abortion Rights,* 47 Hastings Cent. Rep. (2017); Julia Dalzell, *The Impact of Artificial Womb Technology on Abortion Jurisprudence,* 25 Wm. & Mary J. Women & L. 327, 332 (2019).

98 Attorney-General’s Reference no 3 of 1994 [1997] UKHL 31.

99 E. Coke, The Third Part of the Institutes of the Laws of England: Concerning High Treason and Other Please of the Crown and Criminal Causes, 50 (E. and R. Brooke, 1797).

100 Infant Life Preservation Act 1929, s.1 (1).

101 See Romanis, *supra* note 32.

102 Lydia Di Stefano et al., *Ectogestation Ethics: The Implications of Artificially Extending Gestation for Viability, Newborn Resuscitation and Abortion,* 34 Bioethics 371, 377 (2020).

103
*Id.*

104 I. Glenn Cohen and Sadath Sayeed, *Fetal Pain, Abortion, Viability and the Constitution,* 39 J. Law Med. Ethics 235, 237 (2011).

105 Rebecca Cook et al., *Prenatal Management of Anencephaly,* 102 Int. J. Gynecol. Obstet. 304, 304 (2008).

106 A public hearing in the Supreme Court of Brazil found that anencephaly ought not be confused with disability because it was a condition ‘incompatible with life, and because of that, there are no children or adults with anencephaly’. See L. Roberto Barroso, *Bringing Abortion into the Brazilian Debate*, in Abortion Law in Transnational Perspective: Cases and Controversies, 365 (R. Cook et al. eds., 2014).

107 Stillbirth occurs in 32% of cases where anencephalic pregnancies were carried. M. Jaquier et al., *Spontaneous Pregnancy Outcome after Prenatal Diagnosis of Anencephaly*, 113 BJOC 951 (2006).

108 How long the anencephalic newborn survives depends on how much of the brain is missing. Those delivered alive usually do not survive longer than a day. *Id.* at 951.

109 Almost all cases are diagnosed by ultrasound before 14 weeks. There is a 100% detection rate during second trimester sonography: Emre Ekmekci and Servet Gencdal, *What’s Happening When the Pregnancies Are Not Terminated in Case of Anencephalic Fetuses?* 11 J. Clin. Med. Res. 332, 334 (2019).

110 Abortion Act 1967, s.1 (1) (d) as amended by Human Fertilisation and Embryology Act 1990, s.37.

111 Rosamund Scott, *Interpreting the Disability Ground of the Abortion Act,* 64 Camb. Law J. 388, 391 (2005).

112
*Jepson v. Chief Constable of the West Mercia Police Constabulary* [2003] EWHC 3318.

113 Romanis, *supra* note 32, at 109.

114 Grubb, *supra* note 21, at 663.

115 D. Alan Shewmon, *Anencephaly: Selected Medical Aspects,* 18 Hastings Cent. Rep 11, 14–15 (1988).

116 Death is not defined in any Statute in English law but is usually legally determined by a medical diagnosis of brain death: *Re A* [1992] 3 Med LR 303; *Airedale NHS Trust v. Bland* [1993] 1 All ER 821.

117 FL Stat, 2019, § 390.011.

118 22 ME Rev Stat, 2019, § 1598.

119 The American College of Obstetricians and Gynecologists, *Facts are Important: Abortion CareLater in Pregnancy is Important for Women’s Health*, https://www.acog.org/About-ACOG/ACOG-Departments/Government-Relations-and-Outreach/Facts-are-Important-Abortion-Care-Later-in-Pregnancy-is-Important-to-Womens-Health?IsMobileSet=false (accessed Oct. 12, 2019).

120 E.g. those states that place limitations on abortion from 20 weeks gestation: Kansas, North Carolina, Texas, and Wisconsin.

121 Anonymous, *The Myth of Choice,* 170 Ann. Intern. Med. 809 (2019).

122 Born-Alive Infants Protection Act 1 U.S.C. §8 (b).

123 Births and Deaths Registration Act 1953, s.1(1) as amended by the Still-Birth Definition Act 1992, s.41.

124
*Attorney-General’s Reference no 3 of 1994, supra* note 93. A person is still guilty of homicide if they cause injury to a fetus that is born alive before subsequently dying of the injuries sustained.

125 There are (at least) 38 states in which fetal homicide laws have been passed and a number in which women who have experienced the death of their baby are charged with feticide. See Lynn M. Paltrow and Jeanne Flavin, *Arrests of and Forced Interventions on Pregnant Women in the United States 1973–2005: Implications for Women’s Legal Status and Public Health*, 38 J. Health Polit. Policy Law 299 (2013).

126 Supremo Tribunal Federal [Supreme Court] April 12, 2012, ADPF 54/DF at 147.

127 I argue elsewhere that there is no moral continuity between a fetus and gestateling because each entity’s relationship with the female body is different. The same might be said here. Moreover, there are substantial differences in physiology between an entity that is still gestating and one that has been completely born: Romanis, *supra* note 32, at 115.

128 Greasley, *supra* note 86, at 187.

129 To clear, by delivered alive here, I mean an entity that has been delivered from a pregnant person and has made the biological state changes associated with a *complete* birth. See Romanis, *supra* note 90.

130 This is an argument most often made in response to claims about the potential of the embryo.

131 H. Tristram engelhardt, The Foundations of Bioethics 111 (Oxford University Press, 1986).

132 Romanis, *supra* note 32, at 118.

133 A. Stone, Being Born: Birth and Philosophy 3 (Oxford University Press, 2019).

134 Horn, *supra* note 8, at 86.

135 Claire Horn, *Ectogenesis is for Feminists: Reclaiming Artificial Wombs from Anti-Abortion Discourse*, 6 Catalyst: Feminism, Theory, Technoscience 2 (2020).

136 Horn, *supra* note 8, at 113.

137 Dalzell, *supra* note 95, at 341.

138 Roe*, supra* note, 4.7.

139 Margaret Brazier and Emma Cave, Medicine, Patients and the Law, 403 (Manchester University Press, 2016).

140 HL, 1990, Vol 522, c. 1137-114.

141 Son, *supra* note 95, at 223.

142 Tom Lissauer and Graham Clayden, Illustrated Textbook of Paediatrics, 159 (Mosby Elsevier, 2012).

143 Jackson, *supra* note 20, at 74.

144 Department of Health, *Abortion Statistics, England and Wales 2017,*  https://www.gov.uk/government/statistics/abortion-statistics-for-england-and-wales-2017 (accessed Oct. 31, 2019).

145 Tara Jatlaoui et al., *Abortion Surveillance—United States, 2015*, https:/dx.doi.org/10.15585/mmwr.ss6713a1 (accessed Nov. 1, 2019).

146 Eades, *supra* note 71, at 24.

147 Rebecca Cook, *Stigmatized Meanings of Criminal Law*, in Abortion Law in Transnational Perspective: Cases and Controversies, 348 (R. Cook et al. eds., 2014).

148 Elizabeth Chloe Romanis, *Artificial Womb Technology and the Choice to Gestate Ex Utero: Is Partial Ectogenesis the Business of the Criminal Law?* 28 Med. Law. Rev. 342, 373 (2020). https://academic.oup.com/medlaw/advance-article/doi/10.1093/medlaw/fwz037/5680368 (accessed Dec. 18, 2019).

149 Drucilla Cornell, The Imaginary Domain: Abortion, Pornography and Sexual Harassment, 33 (Routledge, 1995).

150
*Id.* at 38.

151
*Id.* at 53

152 P. Singer, Practical Ethics, 126 (Cambridge University Press, 1993); Joel Feinberg, *Abortion*, in Matters of Life and Death: New Introductory Essays in Moral Philosophy, 185 (Tom Regan ed., 1980).

153
*Id.*

154 Cohen and Sayeed, *supra* note 104, at 237.

155
*Id.*

156 Roe*, supra* note 4.

157 Casey*, supra* note 5.

158 Horn, *supra* note 8, at 106 explains that ‘by constructing abortion rights around scientific research on the fetus’s development and increasing potential for personhood, the use of viability timelines for abortion decentralizes the health, needs, and desires of the pregnant woman.’

159 Erdman, *supra* note 2, at 35.

160
*Id.* at 35.

161
*Id.* at 36.

162
*Id.* at 36.

163 In English law, a pregnant person is entitled to refuse any unwanted healthcare intervention even if that intervention was likely to benefit the foetus, e.g. *St George’s Healthcare NHS Trust v. S* [1998] 3 WLR 936; *Re MB (An adult: medical treatment)* [1997] EWCA Civ 3093; *Tameside and Glossop Acute Services Trust v Ch (A Patient)* [1996] 1 FCR 753; *Royal Free NHS Foundation Trust v. AB* [2014] EWCOP 50; *Re AA (Compulsorily Detained Patient: Elective Caesarean)* [2012] EWHC 4378. In the USA, a pregnant person’s right to bodily integrity was recognised in *Re A.C., 573 A.2d 1235* (D.C.1990).

164 Romanis, *supra* note 32.

165 The Pain Capable Unborn Child Protection Act attempted to restrict abortion access from 20 weeks on the grounds that the fetus can feel pain at this point. The Bill received congressional support on four occasions, including 2019, but has yet to receive Senate support. Similar provisions have been enacted at State level.

166 British Pregnancy Advisory Service, *Viability of Extremely Premature Babies,*  http://bpas.org/get-involved/campaigns/briefings/premature-babies/ (accessed Sept. 21, 2019).

167 For example, if we look to Canada, where there is no abortion law since the Supreme Court struck down the 1969 abortion law as incompatible with the Canadian Charter of Rights and Freedoms in R v. Morgentaler 1 SCR 30 (1988), abortion rates appear to be decreasing: Jeanelle Sabourin and Margaret Burnett, *A Review of Therapeutic Abortions and Related Areas of Concern in Canada*, 34 J. Obstet. Gynaecol. Can. 532, 533 (2012), 537–538.

168 Jackson, *supra* note 20, at 72.

169 Northern Ireland (Executive Formation etc.) Act 2019, S.9 (2).

170 This framework passed its final legislative hurdle in Westminster when the Abortion (Northern Ireland) (No. 2) Regulations 2020 was passed on 17th June 2020.

171 The Abortion (Northern Ireland) Regulations 2020, regulation 4.

172
*Id.*, regulation 5.

173
*Id.,* regulation 6; this mirrors s.1 (1) (b) of the Abortion Act 1967.

174
*Id*., regulation 7*.* While this ground for abortion is similar to that contained in s.1 (1) (d) of the Abortion Act 1967, this regulation is far more qualified about what kind of impairment is sufficient to justify termination. It specifies that there must be a ‘substantial risk that the condition of the fetus is such that—(i) the death of the fetus is likely before, during or shortly after birth, or (ii) if the child were born, it would suffer from such physical or mental impairment as to be seriously disabled’. The Abortion Act 1967, in comparison, specifies only that there must be a substantial risk that the child, if born, would be seriously handicapped.

175 Emily Jackson, *Degendering Reproduction?* 16 Med. LR 346, 366 (2008).

176 Sally Sheldon, *The Decriminalisation of Abortion: An Argument for Modernisation,* 36 Oxf. J. Leg. Stud. 334, 362 (2016).

177 Erdman, *supra* note 2, at 35.

178 Horn, *supra* note 135, at 8.

179 Julia Thomson-Philbrook, *Doctor Knows Best: The Illusion of Reproductive Freedom in Canada*, in Fertile Ground: Exploring Reproduction in Canada, 240 (Stephanie Paterson et al. eds., 2014).

180 18 V.S.A., 2019, § 9497.

